# Bridging the Gap: A Review of Pediatric to Adult Transition of Care in Liver Transplantation

**DOI:** 10.1111/petr.14900

**Published:** 2024-12-06

**Authors:** Beverly Kosmach‐Park, Bethany Coyne, Nitika Gupta, George Mazariegos

**Affiliations:** ^1^ Department of Transplant Surgery UPMC Children's Hospital Pittsburgh Pennsylvania USA; ^2^ Department of Family, Community and Mental Health Systems University of Virginia School of Nursing Charlottesville Virginia USA; ^3^ Department of Pediatrics Emory University School of Medicine Atlanta Georgia USA; ^4^ Hillman Center for Pediatric Transplantation, Thomas E. Starzl Transplantation Institute UPMC Children's Hospital of Pittsburgh Pittsburgh Pennsylvania USA

**Keywords:** adherence, adolescence, pediatric liver transplant, transition

## Abstract

**Background:**

With improvements in long‐term graft function and survival, an increasing population of pediatric liver transplant (LT) recipients now require adult care. A process to successfully transition young adults to adult LT centers is supported in the literature with discussions on the rationale for health care transition (HCT), barriers to transition, stakeholder perspectives, and transfer readiness (TR). Results of outcomes studies are difficult to generalize and there remains no standard of care for HCT in LT. Of concern is that the youth's increasing independence occurs during a period of developmental vulnerability, with a threat to graft function due to risk‐taking behaviors, specifically nonadherence, that may lead to rejection, graft loss, and death.

**Objectives/Method:**

The purpose of this comprehensive literature review is to discuss current knowledge, practices, and outcomes of HCT for LT recipients with additional support from literature in solid organ transplant (SOT) and pediatric‐onset chronic conditions literature.

**Results:**

Recent position statements in LT and SOT express a greater awareness of the importance of HCT with broad agreement that reflects a similarity in approach in endorsing HCT as an essential process that should be initiated in early adolescence with TR as a primary determinant of transfer; however, standardization with consistent outcomes measurement is lacking. The literature supports transition as an esential component of care that should be initated in early adolescence with programs that address knowlege, skill‐development, and advocacy. The engagement of all stakeholders in LT is essential to program development.

**Conclusions:**

There is increasing awareness among the multidisciplinary team of the importance and role of the adult provider in extending transitional care into the adult setting as executive functioning skills mature. Outcome measures need to be clearly defined and standardized. Regulatory agency involvement to validate and support the need for TOC programs is crucial and should promote outcomes research for best practice program standardization.

**Abbreviations**
AHA: American Heart Association
AST: American Society of Transplantation
AYA: adolescent and young adult
CHD: congenital heart disease
CKD: chronic kidney disease
CLD: chronic liver disease
EASL: European Association for the Study of the Liver
EF: executive functioning
ESPGHAN: European Society for Pediatric Gastroenterology, Hepatology, and Nutrition
FILFOIE: French Network for Rare Liver Diseases
HCT: healthcare transition
IPNS: International Pediatric Nephrology Association
ISN: International Society of Nephrology
LHS: Learning Health Systems
LT: liver transplant
MLVI: medication level variability index
NASPGHAN: North American Society of Pediatric Gastroenterology, Hepatology, and Nutrition
OPTN: Organ Procurement and Transplantation Network
SNEPT: Starzl Network for Excellence in Pediatric Transplantation
SOT: solid organ transplant
TOC: transition of care
T‐PPACT: Transition Process Planning and Communication Tool
TR: transition readiness
TRA: transition readiness assessment
YA: young adult

## 1 Introduction

End‐stage liver disease in children has been effectively treated by liver transplantation (LT) with 5‐ and 10‐year patient survival at 90% and 88.1% respectively [1]. Although there have been significant improvements in survival over the last 50 years, pediatric LT requires continual monitoring, vigilance, and adherence to care to maintain graft stability and optimize outcomes in every dimension of functioning as pediatric patients age into young adulthood. Since the majority of pediatric LTs are performed in children who are 5 years of age or younger [1], this responsibility begins with parents/caregivers as they work in partnership with the pediatric transplant multidisciplinary team in managing care during childhood. With limited recall and memory, the child's early understanding of transplant care is often associated with the routine of taking medications, obtaining labs, and attending clinic visits under the management of their parents/caregivers and the transplant team. During early adolescence, this responsibility begins to shift and can be supported through a developmentally appropriate and structured transition of care (TOC) process to help youth engage in learning about liver transplant‐related topics and to acquire skills in self‐advocacy and self‐management. Of concern, however, is that the youth's increasing independence occurs during a developmental period of significant vulnerability marked by experimentation and risk‐taking behaviors as concurrent transitions occur in physical, neurocognitive, and psychosocial development. Although adolescents develop decision‐making skills by making mistakes and then gaining knowledge and insight from that behavior, stable graft function may be in jeopardy from this routine process. Experimenting with risk‐taking behaviors, specifically nonadherence to immunosuppression, may result in rejection, related comorbidities, and death. Increased surveillance may be needed during this time to monitor graft function, but adherence to requests for labs and clinic may be postponed or ignored. Outcomes data from pediatric LT centers have reported that 24%–28% of young adults have died within two to five years after transfer to adult hepatology with an additional 8%–15% of patients being lost to follow‐up [2, 3, 4, 5, 6].

Healthcare transition (HCT) has been defined as the “purposeful, planned movement of adolescents and young adults (AYA) with chronic physical and medical conditions from child‐centered to adult‐oriented health care systems” [7]. This definition supports a progressive and individualized approach to healthcare self‐management throughout adolescence that considers the AYA's clinical, psychosocial, and educational needs. Transfer of care is the physical event within transition where the responsibility of providing care shifts to the young adult and to a new setting with an adult team. The literature on transition from pediatric to adult care in chronic conditions is extensive, but the level of evidence is low due to the paucity of studies that investigate the effectiveness of standardized transition interventions through randomized control trials, as well as examining patient recruitment, follow‐up time, and various clinical conditions [8]. Similarly, much has been written about transition for youth who are solid organ transplant (SOT) recipients. The literature to date has highlighted the need and rationale for transition services in SOT, identified barriers to HCT, described patient and family perspectives, and discussed readiness for transfer. Outcomes data from SOT transition programs has been reported; however, results are difficult to generalize due to heterogeneous populations within SOT, limited sample sizes, and a variety of program structures, interventions, and resources without standardization. Consequently, there remains no standard of care for HCT in liver transplantation or other SOT.

### 1.1 Aim

The purpose of this literature review is to discuss current knowledge, practices, and outcomes of transition of care for AYA liver transplant recipients with additional support from the literature in SOT and pediatric‐onset chronic conditions. A greater understanding of the challenges of HCT, current program structures, innovative approaches, educational interventions to promote readiness, and transfer outcomes may contribute to consensus for further development of transition as a standard of care for AYA liver transplant recipients.

## 2 Health Care Transition and Adolescent Cognitive Development

HCT services should begin early and continue throughout adolescence and into young adulthood. This developmental period is crucial as teens explore the skills, knowledge, and abilities required for adulthood. During this time, the adolescent brain undergoes a period of rapid development, particularly in the frontal lobe, which continues into the mid‐twenties. Frontal lobe development is essential for the maturity of executive functioning (EF) skills which impact the understanding of long‐term consequences and impulse control. EF skills are widely recognized as integral to processing speed, planning, organization, working memory, sustained attention, inhibitory control, and cognitive flexibility [9, 10, 11, 12]. As adolescents develop autonomy and independence, immature EF may lead to an increase in risk‐taking behaviors as teens think of themselves as invincible while unable to fully understand the long‐term implications of those risks. The combination of an immature frontal lobe and the need for independence can lead to an increase in risk‐taking behaviors [10]. EF deficits have been recognized in adolescent LT recipients [13, 14] and underline the importance of individualized plans for TOC.

Adolescent transplant recipients face the same developmental tasks and issues as their peers. They struggle with peer pressure and the desire to be “normal.” The need to fit in and peer pressure may increase the risk of medication nonadherence and risky behaviors such as substance abuse [10]. A meta‐analysis examining EF in AYA transplant recipients demonstrated that overall, they had lower EF skills than healthy peers, putting them at high risk for poor decision‐making [11]. These deficits have been correlated with an increase in medication nonadherence and other risky behaviors [14]. Although the incidence of substance use in AYA SOT recipients, particularly alcohol and tobacco, is reported to be similar to or lower than their healthy peers [15], medication nonadherence is particularly high during this developmental period with an incidence of 34% to 70% [16, 17]. As EF matures, targeted education about the outcomes of risk‐taking behaviors on health and graft outcomes, and skill‐building activities to avoid risk‐taking, should continue as essential components of a comprehensive HCT program.

## 3 Health Care Transition and Educational Interventions

Educational interventions are widely recognized as essential to SOT transition programs but lack standardization and methodology. A curriculum that leads to increased knowledge and understanding of transplant health is essential to a comprehensive transition program. This should be implemented through a structured methodology to identify best practice that incorporates transplant care fundamentals, and an approach that recognizes the youth's intellectual capacity and contributory psychosocial and cultural issues. Although using technology is widely accepted by AYAs as a learning resource, a systematic review of transition interventions in chronic illness assessed individualized education programs as the most common educational intervention used in transition programs, followed by group meetings [18]. The majority of studies reported improvements in the youth's knowledge of their chronic condition, self‐management skills, and transition readiness following educational interventions; however, it was difficult to determine a relationship between the type of intervention and subsequent improvement [18].

A scoping review of educational interventions for AYA kidney transplant recipients revealed a wide diversity of educational content, design, and outcomes measurement in kidney transplant education although similarities were seen in regard to having individual education sessions and a transition clinic, and using technology to enhance educational strategies [19]. The authors cited the lack of educational theory in guiding interventions to be a critical gap with the reviewed studies providing minimal details on quality, structure, content, and methods of educational interventions. Few studies incorporated evidence‐based education methods, engaged patients in active learning, or designed interventions to meet the learning needs and abilities of the AYA [19].

Structured educational programs have demonstrated improved knowledge. Engaging in sessions on specific health management skills and participating in interviews with the LT transplant multidisciplinary team for TRA and psychosocial screening resulted in improved transition readiness scores [20]. Similarly, a program consisting of seven 2‐h educational sessions over 2 years resulted in a significant increase in overall medical knowledge, a decrease in nonadherence, and improved readiness in a group of AYA heart transplant recipients [21]. Although education is a fundamental element of the transition process, the authors additionally discuss the importance of assessing confidence, which should also increase over time as the youth has the opportunity to practice and be successful in newly acquired skills [21].

## 4 Supporting Education and Skill Development in LT: A Learning Health System

Learning health systems (LHS), as described by the Institute of Medicine in 2006, are designed to enable continuous improvement with best practices that are ideally embedded in delivery of care to patients [22, 23]. In 2018, the Starzl Network for Excellence in Pediatric Transplantation (SNEPT), the first LHS that focuses on pediatric LT was launched [24]. Through a partnership of multidisciplinary transplant teams, patients, families, and other stakeholders, SNEPT is committed to the development and sharing of best practices in LT. The mission of this LHS is to deliver the best possible care to patients and families by developing achievable solutions to challenges in transplant care through a unified approach using registry data, the latest technology, transplant leadership, and patient advocacy [25].

Providers and family members within SNEPT have identified transition as one of four priority projects for the learning network. Subsequently, a TOC workgroup was formed with additional input from the Patient and Family Voice to develop a prospective data tracking mechanism within the Starzl Data Mart as well as visualizations of key transition milestones within the transfer process [26]. Current goals of the TOC workgroup are to identify predictors of successful transition and to develop evidence‐based guidelines to direct program development and implementation with the goal of sustaining outcomes after transfer to adult providers [27]. SNEPT centers were recently surveyed on existing transition practices through a retrospective study to assess outcomes after transfer to adult care; results to be published. A prospective analysis of outcomes post‐transfer within Starzl Network centers and an ancillary study to design targeted educational interventions based on an assessment of LT knowledge is proposed.

## 5 Recognizing Health Care Transition as a Component of Pediatric Transplantation

An early effort in recognizing the importance of HCT in transplantation was led by the American Society of Transplantation (AST). In 2007, a jointly sponsored international consensus conference was organized to identify fundamental issues related to transition and the adolescent transplant recipient, and to identify processes and strategies to consider in program development [28]. Through expert opinion and discussion, and supported by the existing literature, recommendations for transition in SOT were proposed which included a list of developmental and educational tasks for early, mid, and late adolescence to be discussed during TOC. The domains of self‐concept, family and peers, sexual identity and development, relationship to society (vocational goals), and tasks related to cognitive, moral and affective development were addressed. Areas of skill development and knowledge attainment were recommended that focused on the youth's understanding of their organ disease and need for transplant, the implications of transplant on general health, physical and psychosocial functioning, adherence, and reproductive health and sexuality. Self‐management skills and independence in care should be attained to the best of the AYA's ability. Challenges for the pediatric and adult transplant teams, parents/caregivers, and the teen are discussed with strategies to minimize barriers and promote effective transition. Systems issues are also addressed with health insurance coverage, access to services, and healthcare resources recognized as essential to patient outcomes and in support of TOC program development. Having a joint adolescent and young adult clinic is cited as a model that may lead to greater engagement by the youth with opportunities for role modeling, mentoring, and peer support. Although outcomes assessment is discussed as essential to program evaluation and patient outcomes, specific data points are not provided. Table 1. provides an overview of published consensus statements in SOT and chronic conditions.

**TABLE 1:** Consensus statements in SOT and chronic conditions.

Organization	Goals	Components/guidelines
American Society of Transplantation (AST) Bell LE et al. *Am J Transplant*. 2008; 8(11)	○ Identify fundamental issues related to transition and SOT ○ Identify processes and strategies for program development	○ Developmental and educational tasks for early, middle, and late adolescence ○ Domains: self‐concept, family and peers, sexual identity and development, vocational goals ○ Skills development: disease knowledge, self‐management skills ○ Systems issues: insurance, access to services, resources ○ Strategies to minimize barriers
International Society of Nephrology (ISN) and International Pediatric Nephrology Association (IPNA) Watson AR et al. *Kidney Int*. 2011 Oct; 80(7)	○ Provide a consensus statement on transition from pediatric to adult renal services for patients with chronic kidney disease (CKD)	○ Components of transition identified: transition to transfer, the event of transfer, the transition process, transition/transfer clinic, continuity of care. ○ Interventions or elements of transition rated as essential/desirable for effective transition ○ YA competencies
American Heart Association (AHA) Sable C et al. *Circulation*. 2011; 123(13); John AS et al. *J Am Heart Assoc*. 2022 Apr 5;11(7)	○ Recommend best practices in transitioning YAs with congenital heart disease to adult providers ○ Updated recommendations (2022)	○ Timeline for transition with developmental tasks ○ Recommendations for youth and parental engagement. ○ Anticipatory guidance for reproductive health, genetic counseling, education, vocational choices, exercise, insurance ○ Update endorses previously published fundamentals of transition; psychosocial components and economic impact of inadequate transition added
ESC Association of Cardiovascular Nursing and Allied Professions (ACNAP) et al. Moons P et al. *Eur Heart J*. 2021;42(41)	○ Present a global consensus statement on transition to adult care for adolescents with CHD from 11 international cardiology organizations	○ Supports comprehensive transition of care. ○ Endorses need for provider trainer in CHD ○ Requires effective systems to maintain high quality care into the adult setting
Organ Procurement and Transplantation Network (OPTN); 2018 https://optn.transplant.hrsa.gov/	○ Provide a guidance statement on transition and transfer in SOT	○ Endorses comprehensive approach to TOC that includes the youth and family, partnership with adult providers, transition readiness, formalized policies and procedures, and an updated portable medical summary. ○ Systems issues: prioritize transfer to adult providers within same institution when possible; report transfer status to the OPTN to continue monitoring

The European Society for Pediatric Gastroenterology, Hepatology, and Nutrition (ESPGHAN), the European Association for the Study of the Liver (EASL) Vajro P et al. *J Pediatr Gastroenterol Nutr*. 2018; 66(6)	○ Recommendations for transition of care process for youth with pediatric onset chronic liver disease.	○ Components of TOC: early introduction to the transition process, timing of transition, importance of the multidisciplinary team, transition readiness, pediatric and adult team collaboration, outcomes monitoring.
North American Society for Pediatric Gastroenterology, Hepatology, and Nutrition (NASPGHAN) Vittorio J et al. *J Pediatr Gastroenterol Nutr*. 2023;76(1)	○ Present recommendations for a TOC program for adolescents with CLD and LT.	○ Provides an adapted form of the Six Core Elements of Health Care Transition for CLD and LT. ○ Outcomes assessment: medical outcomes, adherence, health care utilization, QOL, and retransplantation ○ Emphasizes importance of the adult team: early introductions and collaboration between youth and provider, a combined or alternated pediatric/adult transition clinic, and extending transition into adult care

Subsequently in 2011, the International Society of Nephrology (ISN) and the International Pediatric Nephrology Association (IPNA) endorsed a consensus statement on the transition from pediatric to adult renal services that was developed by both pediatric and adult nephrologists [29]. Similar to the AST recommendations, the statement describes transition as an individualized process that should focus on developing self‐management skills. The consensus statement presents five broad components of transition: transition to transfer, the event of transfer, the transition process, transition or transfer clinic, and continuity of care. Each category includes a list of interventions or elements that are rated as essential or desirable for effective transition and program development. Although these recommendations are specifically targeted to adolescent renal transplant recipients, they are readily applicable to transition in SOT.

In the same year, the American Heart Association (AHA) published a scientific statement on best practices in transitioning AYAs with congenital heart disease to adult providers [30]. Each recommendation for best practice is classified according to the size of its treatment effect (benefit vs. risk, no benefit, harm) and the certainty of the treatment effect. All recommendations were determined to be Class I with the benefit greatly outweighing the risk of that intervention; however, the level of evidence for the majority of recommendations was low (Level C) due to support by consensus opinions, standards of care, and case studies rather than clinical or randomized trials. A timeline for transition is detailed by age groups with specific developmental tasks for the AYA with congenital heart defects. Psychosocial outcomes and family dynamics, particularly parental involvement during transition, are presented with recommendations for both youth and parental engagement in the transition process. This comprehensive approach to best practice also includes anticipatory guidance in regard to reproductive health, genetic counseling, education, vocational choices, exercise, and insurance. The AHA Statement endorses the fundamentals of transition that are now generally accepted in the field including defining transition as a planned process that should be individualized, introducing transition at an early age, engaging the family in the transition process, participating in a formal education curriculum to understand their chronic condition and medical care, providing flexibility for the timing of transfer that reflects the AYA's ability to achieve autonomy of care to the best of their ability, and preparing and implementing a coordinated transfer process in collaboration with adult providers. TOC for the adolescent heart transplant recipient is not specifically addressed in this statement, but guidelines could be adapted for this population based on this document. An update of these recommendations was published by the AHA in 2022 which continues to support transition programming in CHD programs, while acknowledging the barriers and challenges to program implementation [31]. The update provided additional psychosocial components for inclusion in transition programming including social determinants of care, well‐being, and neurocognitive skills. The economic impact of inadequate transition, which has not been extensively studied, is also recognized citing the impact of loss to follow‐up on health care utilization and costs, and a diminished quality of life. This recommendation underlines the importance of the economic perspective of transition that should be factored into justification statements for TOC program development. A family‐centered approach, effective communication, and a coordinated effort among all stakeholders are endorsed as key considerations of program development.

Transition of care in CHD was further supported through a recent global consensus statement developed by 11 cardiology organizations [32]. This statement describes issues and clinical practices for transition of care, similar to those posited earlier by the AHA. While continuing to endorse comprehensive TOC, this work emphasized the need for qualified adult providers with training in CHD, and the importance of systems to be in place to ensure that high‐quality care is continued and maintained through transfer into adult care. This need is echoed in the SOT transition literature in regard to inadequate knowledge and training in congenital conditions, adolescent medicine, and post‐transplant care management [16, 33, 34, 35].

From the transplant perspective, the Organ Procurement and Transplantation Network (OPTN) published a guidance statement on transition and transfer for pediatric SOT recipients that was developed in response to adverse patient outcomes associated with transfer to adult providers including nonadherence, loss to follow‐up, and graft loss [36]. Provider responsibilities are proposed for the pediatric and adult transplant teams based on data from an online survey of transition and transfer practices of UNOS centers that are consistent with the literature. The statement endorses a planned and comprehensive approach to transition that includes the family as well as the youth, an established partnership with adult providers, a routine assessment of transition readiness, formalized policies and procedures for transfer, and an updated portable medical summary. Similar to the CHD recommendations [32], the OPTN statement favors establishing partnerships with adult providers well in advance of transfer while prioritizing transfer to adult transplant providers within the same institution whenever possible to maintain quality of care into the adult setting. Patient transfers between transplant centers should be communicated to the OPTN so that health information sharing is continued [36].

Pediatric liver disease and transplant organizations have published position papers to endorse TOC in these populations and provide recommendations for program development and implementation. The European Society for Pediatric Gastroenterology, Hepatology, and Nutrition (ESPGHAN), the European Association for the Study of the Liver (EASL), and North American Society for Pediatric Gastroenterology, Hepatology, and Nutrition (NASPGHAN) proposed a transition process for youth with pediatric‐onset chronic liver disease (CLD) or AYA LT recipients through a standardized and multidisciplinary approach [37, 38]. Program components identified as essential to TOC include an early introduction to the transition process and timing, the importance of a multidisciplinary transition team, transition readiness, pediatric and adult team collaboration, and outcomes monitoring. The recommended transition program structure is based on the Six Core Elements of Health Care Transition with adaptations for LT and CLD [38]. The importance of the adult team is emphasized through recommendations of early introductions and collaboration between the youth and providers prior to transfer, a combined or alternated pediatric/adult transition clinic, and extending transition into adult care until the YA is fully integrated into the adult setting [37, 38].

## 6 Barriers and Challenges in Transition: Opportunities for HCT Program Development

The barriers and challenges associated with TOC are well‐described in the literature and are often inter‐related and complex. Four categories of barriers, as identified by adult nephrologists, were reported including lack of available resources, patient skills and social support, provider education in adolescent health and complex congenital disease, and collaboration with the pediatric team [35]. Provider education is of concern for pediatric providers in transferring AYAs because they perceive the adult provider to lack training to care for pediatric patients with complex conditions. They also believe that adult providers do not want to accept these patients because of the increased burden of time and effort required for their care [35]. Identified barriers to transition in LT are similarly described and categorized by patient, provider, and systems‐level perceptions [33]. The most commonly cited barriers at the patient level included willingness to participate in transition, failure to show up for the first appointment, and patient reluctance. Provider education in congenital disease and difficulty identifying an adult provider were provider level barriers, and resource‐related issues were most commonly cited at the systems level [33].

### 6.1 Family Barriers

The inter‐relationships of stakeholders within the transition process are often discussed as critical barriers and merit further discussion as an important area to more fully understand. The quality of the parent–child relationship is an important factor in transition and interestingly, may act as a barrier or a facilitator during the process. Family cohesiveness, defined as the sense of unity and togetherness in the family, is identified as a significant predictor of transition readiness (TR) in a study of adolescents with chronic kidney disease (CKD) [39]. The authors suggest that adolescents who have the support of a cohesive family structure will be better equipped to manage their care independently. This finding speaks to the importance of family assessment within the transition process to provide information about the transition process for both the youth and parents, to set goals for each as self‐care is gradually acquired by the AYA, and to intervene with strategies to improve family cohesiveness.

In addition to the importance of family cohesiveness, the youth's reliance on parents as a source of knowledge was demonstrated in a study of 160 youths (age range 6–16 years; mean 12.2) with chronic conditions, including kidney and liver disease and supports the importance of including parents in the transition process [40]. This cohort preferred to obtain information about their health care from their parents, followed by health care providers and the Internet, although differences were seen by age with older teens preferring to use the Internet. As a young adolescent, it is understandable that youth rely on their parents for information, but this preference may shift as they attain self‐management skills and greater independence in middle and late adolescence. The study also reported that AYAs with the highest medication adherence preferred parents as their information source, while those who chose health care providers had the highest scores in self‐efficacy and TR [40]. Building the foundation of medical knowledge and self‐management skills with parental support during the early phase of transition may contribute to improved TR as older teens begin to rely more on health care providers. Parents should have access to accurate information about their teen's health care management that reinforces education from providers [40].

Parenting styles that support the gradual shift of responsibility for care from the parent to child rather than one of overprotection and an unwillingness to engage in the process, may promote a successful transition to adult care [28, 41]. Literature in heart transplant has demonstrated that parental support during adolescence has a positive association with quality of life and patient‐reported health, while decreasing depression and loneliness [42]. Although parents of heart transplant recipients in transition exhibited feelings of anxiety, their child was more inclined to convey apathy [43]. Parental anxiety and overprotection during the transition process may transmit feelings of distrust between the youth and parent, which may promote continued passivity and decrease the YA's engagement in transition [43]. Over‐protective and over‐involved parents and the inability of the parent to “let go” are often cited as barriers that affect the AYAs progress toward autonomy [35, 41, 43, 44, 45]. Although family involvement is seen to be related to improved TR, the level of involvement needs to be assessed, setting expectations for both the parent and adolescent. The multidisciplinary team plays an essential role in assessing parenting style and its effect on autonomy development throughout adolescence. The appropriate level of parental involvement during TOC should be determined and may range from engaging uninvolved parents for increased support or limiting participation by overinvolved parents. Starting at around 13 years of age, AYAs should meet privately with providers for at least a short period of time during appointments to help them develop self‐advocacy skills and to promote independence [37, 41, 46, 47, 48].

### 6.2 Provider Barriers

The attachment between the pediatric provider and patient/family is commonly cited as a significant barrier in approaching transition of care [28, 33, 34]. For many families, this relationship began with the child's diagnosis and developed over the years through times of crisis as well as stability, creating a strong bond and trusting relationship between the parent/child and pediatric provider. In focus groups, parents describe a closeness that is similar to what is felt within families, and consequently transition represents a loss of their relationship with pediatric providers [49]. With this change in the patient‐provider relationship, a theme of fear has been expressed by youth through reluctance to leave pediatric care and increased anxiety in not knowing the adult team, health care system, and the myriad of logistical issues that arise with transfer of care. Parental anxiety is related to relinquishing control and not feeling valued by the adult team for their knowledge and experience in their child's care [47]. Similarly, LT youth reported moderate concern about transfer to adult care, with the greatest concerns identified as the adult team not being as caring, leaving their pediatric providers, and having to meet new caregivers [50].

A survey of pediatric gastroenterologists reported parent (81%) and patient (74%) attachment to pediatric providers as the most frequently cited barriers to transfer to adult care [51]. Additional attachment‐related barriers included the provider's attachment to the patient or family (56%), and the parent's (54%) and patient's (46%) attachment to the institution or practice [51]. Pediatric providers may also inadvertently add to this barrier through an attitude of ambivalence or negativity about transfer to adult care that compounds the youth's and family's anxiety about the change in care. This may lead to a delay in youth and parental engagement in the transition process and developing a relationship with the adult team [38, 52, 53]. The relationship barrier may be mitigated through a structured transition program which supports ongoing and early education about the process of transition, an early introduction to the adult team, and interdependence between the teams to support the YA in transition [51, 54, 55].

The need for a multidisciplinary approach to a collaborative and integrative transition process is well‐supported in the literature [54, 56, 57] and also recommended in LT [33, 37, 38]. Patient, provider, and systems barriers to HCT are cited as being best addressed through a multidisciplinary approach to transition planning that incorporates effective partnerships and collaboration between pediatric and adult providers [38]. This multidisciplinary collaboration is also supported by the Transition Working Group of the French Network for Rare Liver Diseases (FILFOIE) who endorses consultations with the adult and pediatric providers in a joint clinic when geographically possible, or alternated consultations for a period of time before transfer [58]. Joint consultations are described as a means to promote effective communication between the teams for clinical updates, psychosocial concerns, and TR, and should extend to the youth including initial contact that is “as humane and personal as possible.” [58] The Transition Group endorsed effective communication, particularly through phone calls, to confirm contact information, demographics and basic clinical information, and to schedule the adult clinic appointment [58].

### 6.3 Sytem Barriers Systems Level Barriers

Barriers at the systems level also pose challenges to effective TOC and are associated with the differences between the settings, approach, structure, and resources in pediatrics versus adult care (Table 2). Systems barriers may also be further influenced by inter‐related barriers, such as how the established parent‐pediatric provider bond affects the parental role in the adult setting. In an assessment of their perceptions of the transition process, adolescent LT recipients and their parents reported system levels concerns including being admitted to the adult facility, establishing care with new providers, level of caring by adult providers as compared to the pediatric team, and the quality of care in the adult setting [50]. Parents reported wanting to know more about the transition process and transfer, but interestingly, preferred to receive this information from the pediatric team. The authors remarked that this preference may reflect that parents' strong attachment to the pediatric team can affect the transition process [50].

**TABLE 2:** Systems differences between pediatric and adult liver transplant care.

Systems characteristic	Pediatric LT/hepatology care	Adult transplant hepatology
Focus of care	Child development and growth within the context of LT Monitor transplant comorbidities	Maintenance of graft function Monitoring for long‐term comorbidities related to LT and immunosuppression
Approach to care	Family‐centered care with shared decision‐making between parents and providers. Patient may have a more passive role even into their teens. Comprehensive, psychosocial approach to care	Patient‐centered care with collaboration between patient and provider Problem‐based approach Focus on LT and comorbidites
Transplant Setting	Usually a free‐standing pediatric institution Patient is within the same LT department from diagnosis through young adulthood. May be affiliated with an adult center for transition/transfer	Independent adult LT program May collaborate with one or more pediatric LT centers for transfers In the US, insurance is a significant factor in determining center choice for adult care
LT Providers	Follow‐up with Transplant Surgery for at least 1 year after LT, then as needed. Routine follow‐up with pediatric Hepatology or GI Parents often regard these physicians as their primary care providers	Adult Transplant Hepatology or GI Transplant Surgery consulted as needed.
LT multidisciplinary team	Usually involves an extensive multidisciplinary team: APPs, Social Work, Psychology, Pharmacy, Child Life, and various therapies. Consults/referrals are made within the team. Care conferences and multidisciplinary team meeting reviews are common.	Less availability and support for multidisciplinary providers within the LT team. Limited access for routine or acute assessment from Social Work or Psychology/Psychiatry Assessment of need often deferred to the patient's Primary Care Provider
Availability of Care Coordination	Usually available in pediatrics Transition coordinator used to facilitate process A variety of resources are usually available based on need and criteria	Rarely available in adult care Patient needs to self‐advocate for resources/support Less resources for additional care needs, mental health
Appointment length	Length of appointment is tailored to patient need; longer appointments are common Additional referrals often coordinated with routine appointments for patient convenience and scheduled by LT team	Usually brief based on acuity Appointment often conducted by one provider Referrals are scheduled as needed; patients coordinate appointments
Time alone with provider	Infrequent; patient seen with caregiver Teens should meet the provider alone for at least part of the appointment during transition Patients should be instructed about the changes in privacy and confidentiality that occur at 18 years of age.	Legally required for patients > 18 years of age May consent to others being present during appointment

Self‐advocacy	Initially has a passive role Self‐advocacy skills should be nurtured in the early teens may by directly asking youth about their care. Providers may interact more with caregivers than patient regarding care and decision‐making	Self‐advocacy skills are essential to establish a collaboration relationship with the adult team. Support from family and friends may be helpful for some patients.
Self‐care skills	Passive role in self‐care until able to take more responsibility Parents usually manage and facilitate medication refills, appointments, follow‐up requests.	Active role in self‐care: medication management, appointments, follow‐up, communication with team May need additional support depending on abilities
Communication	Communication is usually between the parent and team only. Over time, information is usually relayed by the parent to the teen At 18 years of age, communication must be with the patient although they can consent to their parents being involved.	Adult actively communicates with the team
Adherence to care	Frequent reminders and communication to improve/maintain adherence Strategies actively utilized with patients and parents to maintain adherence. Mental health (Transplant Psychology, Social Work) and Child Protective Services are available for significant nonadherence that increases the risk for graft dysfunction/failure and comorbidities.	Adherence to recommendations, treatment, and follow‐up are expected. If patients choose not to follow provider recommendations, no legal options are available Mental health services may be recommended to address nonadherence risks

*Note:* Adapted from: Got Transition. System differences between pediatric an adult health care. Accessed March 6, 2024. https://www.gottransition.org/resource/?system‐differences‐between‐pediatric‐and‐adult‐health‐care; King LY, Kosmach‐Park B, Parish A, Niedzwiecki D, Jackson WE, Vittorio JM. Current approach to health care transition and integration into adult care for pediatric liver transplant recipients: A call for partnership. *Clin Transplant*. 2023; 37: e14990. https://doi.org/10.1111/ctr.14990.

## 7 A Structured Transition Process for Transfer From Pediatric to Adult LT Services

Since the initial call for transition services in the 1990s, various transition programs and resources have been developed. Got Transition, a program of the National Alliance to Advance Adolescent Health, provides a national resource center with the aim to provide evidence‐based resources for providers, youth, and caregivers to improve the transition from pediatric to adult heath care [54, 59]. The Got Transition implementation guides are individualized into three approaches to transition: transitioning to an adult health care clinician, transitioning to an adult approach without changing clinicians, and integrating young adults into adult health care. In the first approach, youth are prepared for transition by pediatric providers and eventually transferred to new adult providers. Some transition programs are integrated with the youth continuing care with the same providers while transitioning to an adult approach to care within the same practice. The third model focuses on the integration of the youth into adult health care systems [60].

In a survey of one of the earlier TOC programs in LT, pediatric and adult team members endorsed the need for changes in practice to better support youth as they prepared for and transferred to adult providers. Team members recommended implementing organizational changes to redesign the transfer process, incorporating a transfer checklist, and adopting a long‐term care approach to TOC prior to transfer that would assess readiness for transfer and teach health care management skill [61]. These findings reflect early TOC programs which were concerned about gaps in care as the YA transferred to adult care but lacked a more comprehensive and structured approach. Continued program development with resources and templates to be used during the transition process in chronic illness populations, have provided a framework for TOC programs in LT and are endorsed within the community [38]. A multidisciplinary team approach with collaboration throughout the transition process by both pediatric and adult providers is necessary for program development. Team composition will vary based on center size, staffing, and resources, but in addition to physician providers, the system should strive to support nursing, psychology, social work, and pharmacy assessments and interventions into the adult setting [38].

In contrast to a structured TOC program, patients who were not transferred and continued with the same providers and institution were reported to have good long‐term outcomes at 5‐ and 10‐years (100% and 93% respectively) after reaching adulthood at age 18 [62]. Although 11% of LT recipients were described as nonadherent and 14% had a psychiatric diagnosis, no associations were reported between adherence, rejection, mental health diagnosis, and survival. The authors believe that avoiding transfer to a new adult team and maintaining the same team as the young adult continues to mature contributes to favorable outcomes [62]. Interventions for transitioning “in‐place,” a methodology for an adult approach to care within the institution, or how the transplant team supported youth in achieving self‐management skills and independence were not addressed. The framework for transition within the same setting, as presented by Got Transition, should still embrace a process for adjusting to the adult approach to care, and requires similar interventions including routine skills assessment, a collaborative plan of care, resource identification, ongoing education based on cognitive skills, and a discussion of how communication, privacy and confidentiality will be approached [54].

Comprehensive multidisciplinary approaches to transition for youth with LT vary by institution depending on available resources and structure. The most common approach in this patient population involves transitioning youth from pediatric to adult providers, either within the same health system, or to an external system. The Six Core Elements of Health Care Transition is presented as a broad framework for transition process that can be adapted for all chronic conditions and supported in the transplant literature [20, 33, 38, 63]. The elements are presented in detail through implementation guides [54] and can be used to develop, strategize, and execute transition programs for adolescent LT recipients. Each of the Got Transition Core Elements will be discussed in relationship to the unique needs of LT programs.

### 7.1 Transition Policy

The development and implementation of a transition policy specific to the LT practice is the first step of a structured transition program. The policy should be formed with input from providers, youth, and their caregivers and presented during enrollment in TOC, ideally at 12 to 14 years of age [54, 63]. Comprehensive policies should include information about how the pediatric LT team approaches transition and transfer, the roles and responsibilities of the LT multidisciplinary transition team, changes in confidentiality and privacy in reaching legal age, and the team's recognition of cultural preferences and intellectual capacity. Institutional mandates regarding “aging out” should also be outlined. For providers receiving youth into their care, the adult team policy should outline the adult approach to care and how the YA will be integrated into their system. In a recent survey of centers caring for youth who have undergone LT, pediatric centers were more likely than adult centers to have a transition policy (60.2% vs. 39.2%) and adult centers who were affiliated with a pediatric system were more likely to have formal transition policy [33]. The transition program structure described by Shapiro et al. provided a transition policy to all LT patients 13 years of age and older [20]. The transition policy can also be included within the after‐visit summary, through a message on the patient portal, the clinic website, or as a handout distributed during clinic [64].

### 7.2 Tracking and Monitoring

A key component for transition program success is establishing a mechanism for tracking and monitoring individuals as they prepare for transfer and a system for documenting outcomes. Although there is agreement that the use of electronic medical records (EMR) can help facilitate individual metrics and monitoring, there is a paucity of research in tool development, feasibility, or outcomes for TOC. Outcome monitoring in SOT has typically relied on the Organ Procurement Transplantation Network (OPTN) data which can be helpful for tracking long‐term outcomes, but data are not captured specifically about transition services, details of the transfer process, or timing of transfer, thus limiting the usability of documentation. In response to the limited availability of standardized tracking tools for transition, a transition process planning and communication tool (T‐PPACT) was developed that incorporates the Six Core Elements of Transition to be used throughout the transition process by the multidisciplinary team to facilitate communication, collaboration, and coordination of care [65]. The tool was piloted in 38 subspecialty clinics through 10 professional roles at a large pediatric health system and included liver, kidney, and heart transplant recipients. Study results reported willingness among providers to use an EMR‐based transition tool with the highest acceptance by nurses. The study reported a significant increase in HCT documentation following implementation of the T‐PACCT which suggests that the tool may contribute to transition program development [65].

### 7.3 Transition Readiness

As the national call for HCT services in transplantation and publication of the previously discussed position statements and guidelines, subsequent reports on HCT programs in LT reflect a variety of interventions, resources, and outcomes. Transition readiness assessment (TRA), as a component of HCT, is widely recognized as an essential element of preparation to assess gaps in knowledge and skill development, and to prioritize and direct interventions to mitigate these gaps. Got Transition recommends assessing TR in early adolescence and by age 14. Several TRA tools have been discussed in the transition literature; however, most of these are general measures of readiness and not specific to SOT. The American Society of Transplantation (AST) has developed TRA templates and check lists that are specific to youth in transition from pediatric to adult transplant providers; reliability and validity have not yet been confirmed [66]. See Table 3 for a summary of TRA instruments.

**TABLE 3:** Transition Readiness Assessment Tools.

Transition Readiness Tool	Description	Assessment domains
Got Transition ○ https://www.gottransition.org/ ○ https://gottransition.org/6ce/?leaving‐readiness‐assessment‐youth White PH et al. *Pediatrics*. 2018; 142(5)	○ 23‐item self‐administered questionnaire ○ Scoring: 3‐level scale (no, I want to learn, yes) ○ 2 items on transition importance and confidence ○ Scoring: not important (0) to very important (10)	○ Health and healthcare knowledge (20 items) ○ Medication knowledge (3 items) ○ Transition importance and confidence (2 items)
STARx Questionnaire https://www.med.unc.edu/transition/transition‐tools/trxansition‐scale/ Ferris M et al. *J Pediatr Nurs*. 2015; 30(5); Cohen SE et al. J Pediatr Nurs. 2015; 30(5)	○ 3‐domain, 18‐item self‐administered questionnaire ○ Scoring: 5‐level Likert scale in 3 versions: patient in pediatric setting, patient in adult setting, parent form ○ Web‐based data entry system or hard copy ○ Reliability and validity established	○ Medication management ○ Provider communication ○ Engagement ○ Disease knowledge ○ Adult health responsibilities ○ Resource utilization
The TR_x_ANSITION Index https://www.med.unc.edu/transition/transition‐tools/starx‐questionnaire/ Single sheet handouts related to each domain are available for additional patient education. https://www.med.unc.edu/transition/transition‐tools/copy_of_educational‐handouts‐for‐trxansition‐indextm Cantú‐Quintanilla G et al. *J Pediatr Nurs*. 2015; 30(5)	○ 10‐domain, 32‐item questionnaire administered by a trained professional ○ Scoring: No knowledge or skill attainment (0), some knowledge or skill attainment (0.5) or adequate knowledge or skill mastery (1) ○ Web‐based data entry system or hard copy ○ Reliability and validity established	○ Type of illness ○ Rx/Medications ○ Adherence ○ Nutrition ○ Self‐management skills ○ Issues of reproductive health ○ Trade/school ○ Insurance ○ Ongoing support ○ New health care providers
Transition Readiness Assessment Questionnaire (TRAQ) https://www.etsu.edu/com/pediatrics/traq/default.php Wood DL et al. *Acad Pediatr*. 2014; 14(4):415‐22; Johnson K et al. *J Pediatr Nurs*. 2021; 59:188‐195	○ Version 5.0: 20‐item, 5 domain patient‐reported assessment ○ Scoring: 5‐level Likert scale ○ Online questionnaire or hard copy ○ Reported to have good internal reliability and criterion validity	○ Managing medications ○ Keeping appointments ○ Tracking health issues ○ Talking with providers ○ Managing daily activities

Pediatric Transition Portal, American Society of Transplantation (AST) https://www.healthytransplant.com/education/specialty‐resources/peds‐transition The Transition Toolkit contains templates to be adapted to individual transplant centers based on patient population and resources. ○ Transition readiness assessment tools (qualitative interview and checklist) ○ Transition Action Plan ○ Parent Action Plan ○ Transfer Summary forms.	○ Templates developed for early, middle, late adolescence. ○ Higher scores associated with higher readiness competency ○ Tracking scores longitudinally demonstrates improvement or gaps in readiness ○ Reliability and validity not yet established	○ My Transplant ○ My Medications ○ Adherence ○ Risk‐Taking Behaviors ○ Managing my Health: What I Do to Stay Healthy ○ Self‐Advocacy ○ My Reproductive Health ○ Going to School ○ My Support System ○ How I Feel about Myself ○ Paying for my Healthcare
The Allocation of Treatment Responsibility Scale (ATR) This instrument measures the distribution of treatment tasks across family members of kidney transplant recipients, ages 7‐18 years. Pai AL et al. *Pediatr Transplant*. 2010;14(8); Pai AL, Drotar D. J *Pediatr Psychol*. 2010;35(4)	○ 18‐item questionnaire ○ Child and parent versions ○ Scoring: 4‐level Likert scale ○ Online questionnaire or hard copy ○ Strong internal consistency; further studies needed to confirm validity.	○ Medications ○ Clinic attendance ○ Lab attendance

Encouraging results from TRA and associated interventions have been reported. A quality improvement initiative for pediatric LT patients reported positive results through the implementation of a TR skills program. Improvements in medication adherence and attendance at the first clinic visit with adult providers were demonstrated by those participating in the program [67]. TR was a key strategy in another transition program using the Six Core Elements of Transition with targeted interventions demonstrating an improvement in TRA scores [20]. Transition interventions included completion of a TRA tool at the initial transition visit and the final pediatric visit prior to transfer, an assessment by a psychologist to identify potential psychosocial barriers to transition, education support to identify skill and knowledge gaps and acquisition, and additional sessions with other transition team members to address various barriers in transition. The significant improvement of TRA scores (+28.7%; *p* = 0.009) supported the use of the Six Core Elements of Transition as a framework for transition program development and quality metric tracking [20].

Transition readiness in LT, including confidence in asking questions about their healthcare, responsibility for taking medications, and knowledge of the healthcare effects of drugs and alcohol, was significantly improved following participation in a TOC program [46]. Although there were no differences in the incidence of rejection or loss to follow‐up between intervention and non‐intervention groups, the program's individualized approach to transition skills resulted in improvements in transition‐related competencies, decreased patient anxiety about transfer, and increased patient satisfaction [46]. Similar results, including an increase in knowledge, confidence in their ability to transition, and improved adherence, were reported in a group of heart transplant recipients who participated in a structured transition program [21]. The authors suggested that communication and collaboration between all stakeholders is essential with ongoing consultation between the pediatric and adult providers post‐transfer.

Although individualized interventions are demonstrated to improve TR, an assessment of the AYA's executive functioning can further identify the best approach to achieve transition skills. The relationship between TR and EF skills in adolescent transplant recipients emphasizes the importance of individualized educational plans based on an assessment of EF skills and the YA's responsibility in improving readiness. TR is associated with two domains of EF: metacognitive skills, which include planning and organizational skills, working memory, initiation, and monitoring; and behavioral regulation, which is the ability to manage and control behaviors and emotions [14]. A study of caregivers' perspectives of their child's TR and EF following liver, kidney, or heart transplant reported that less TR and less AYA responsibility was significantly correlated with EF deficits. The authors suggested that although many transplant centers rely on chronological age as a determinant for transfer, EF should be considered as an indicator for TR and should be assessed in regard to the youth's responsibility for their healthcare [14]. This association between TR and EF was also cited in a similar study from the youth perspective which reported that adolescent transplant recipients who perceived themselves as mostly or completely ready for transfer had significantly higher EF than those who reported not being ready for transfer. These youth had achieved greater responsibility in managerial healthcare tasks including scheduling and insurance knowledge [68]. Although the majority of youth in the study reported being not at all or somewhat ready for transition, all AYAs had significantly greater responsibility for routine tasks when compared to the managerial tasks. The authors discuss the importance of a hierarchical attainment of healthcare tasks that progresses from routine tasks in taking medications, obtaining labs, having an explanation for their medical condition, attending appointments, and communicating with their providers to higher‐level managerial tasks. It is suggested that the acquisition of managerial skills, rather than routine tasks, is related to the AYA having a better perception of TR and recognized EF as an essential component in achieving self‐management skills [68].

From the provider's perspective, adult transplant hepatologists reported that only 46% of youth entered adult care with adequate knowledge of their condition [16]. Additionally, they cited deficits in various elements of TR including poor adherence to medications, obtaining labs, and requested follow‐up (71.5%); inadequate knowledge of how risk‐taking behaviors affect health (55.8%); and limited self‐management skills (54%) and knowledge about their medical and surgical history (51.6%) which were considered to be the greatest barriers to optimal care after transfer [16]. Interestingly, adult providers who participated in a formal transition program (15.5%), cited fewer knowledge gaps in regard to the youth's understanding of the impact of LT on their daily lives, fitness, and sexuality suggesting a positive effect of transition interventions.

Inadequate knowledge regarding the transition process and medical information about their healthcare was a theme that emerged through focus groups with LT recipients who had transferred care [69]. Youth were interviewed about their current care, experiences with transition, and the use of technology to support transition. Although basic information about their healthcare had been acquired, YAs suggested that this be repeated within the framework of transition and the perspective of their care in the adult setting. Youth expressed concern that they did not know whom to contact for assistance in obtaining information about their health. Additionally, since these youth were transferred with limited preparation at a specific age (16–18 years) rather than when transition readiness was achieved, a theme of abandonment or being “dumped” by the pediatric team was suggested [69]. YAs also thought that mobile phone technology could provide information and contacts, and had an interest in exploring apps that would support transition information needs [69].

### 7.4 Transition Planning

Transition planning encompasses preparation and decision‐making related to transfer. Youth and caregiver education should be related to areas jointly identified as priorities by both the pediatric and adult LT teams, and knowledge deficits identified from the TRA. Considerations regarding decision‐making, insurance coverage, and community resources should also be explored in preparation for transfer with confirmed accessibility to needed resources. The timing of transfer should be discussed early in the planning stage and should be individualized, flexible, and in agreement with youth and providers. Communication and collaboration among all stakeholders is essential and includes all pertinent medical records, additional referrals to adult clinicians, and an updated medical summary. Medical transfer summary templates for SOT recipients, which can be adapted for center‐specific needs, are available on the AST website [66]. Another helpful strategy during transition planning includes participation in routine planning meetings with both teams to update individual patient progress and to assess overall program process improvement.

### 7.5 Transfer of Care

Transfer is not merely an administrative or chronological event, but as previously discussed, should be individualized based on preparation, readiness, and willingness of youth, caregivers, and adult providers to engage in care during a time of patient stability. Joint visits where youth and parents meet with both teams during a visit prior to transfer or through virtual “Meet and Greets” should be considered [70]. Communication between teams during this time is essential, ensuring the transfer visit with the adult team occurred and clarifying any information needed.

### 7.6 Transfer Completion

HCT does not end after transfer but extends into adult care until the YA has achieved optimal EF to the best of their ability. Initial care in the adult setting should be consistent and regular. It is recommended that youth have a dedicated lead transplant hepatologist for at least 1 year post transfer and have more frequent follow‐up, every 3 to 6 months, for the first year post transfer [46]. The need for multidisciplinary team involvement remains essential although these resources may be limited in the adult setting [38, 71]. Got transition has tools and resources for the adult care team to continue to support youth as they adapt to adult‐oriented systems of care [59].

## 8 Outcomes of HCT Programs

A systematic review of transition studies in chronic health conditions reported statistically significant positive outcomes in 65% of the studies with improvements in health, the transition experience, decreased barriers to transition, resource utilization, and/or adherence through structured transition programs [72]. As TOC programs develop in LT, outcomes monitoring is vital to program evaluation to assess the effect of interventions on maintaining patient survival and graft function. Various outcome measures have been proposed in the chronic illness literature, but definition and measurement have lacked consistency. Suggestions for outcomes to be monitored include disease‐specific measures, clinic attendance, hospital admissions pre‐ and post‐transfer, relationship with the adult provider, self‐management skills (adherence, health care utilization), assessment of quality of life, vocational outcomes, and patient and family satisfaction [73, 74]. In LT, outcomes monitoring is recommended to include graft function (liver function tests), episodes of biopsy‐proven acute and chronic rejection, graft and patient survival, and measures of adherence related to immunosuppression medications [71]. The NASPGHN position paper recommends assessing medical outcomes (patient and graft survival, graft health, and complications of immunosuppression), adherence (medication and patient follow‐up), health care utilization (ED visits, hospitalizations, length of stay), health‐related quality of life (satisfaction, vocational outcomes, independent living, mental health, and substance use), and retransplantation [38]. Standardizing the outcomes to be monitored will help identify the effects of specific HCT interventions associated with maintaining LT health and achieved self‐management skills.

There is a growing body of literature on transition outcomes in LT; however, assessing outcomes following transfer to adult transplant hepatology is challenging and results are difficult to generalize due to program variability, inconsistencies among existing transition programs, and limited sample sizes (Table 4). Good long‐term outcomes were reported in a retrospective study of young adult LT recipients (*n* = 137) who were transferred to adult care from 1994 to 2012 in the United Kingdom [75], although this is discussed in regard to clinical data and outcomes versus transition readiness. The median age of transfer was 18.6 years, median follow‐up time since transfer was 5 years, and 1‐, 5‐, and 10‐year patient survival after transfer was 98.4%, 93.7%, and 89.9% respectively [75]. There were inconsistencies in transition interventions with the earlier patients being prepared for transfer by the pediatric hepatologist who confirmed when the youth was “ready” for transfer. The pediatric hepatologist and a nurse attended the first adult clinic with the patient who would then be integrated into the adult setting. A transition program was initiated in 2009 with a comprehensive program that includes educational programming and a transition nurse specialist who works across both the pediatric and adult settings to continue to care for the YA in the adult setting until age 25. Over 30% of this group participated in the newer transition program, although none participated in the entire program given the time of its introduction. Knowledge of their condition and medications, self‐management skills, self‐advocacy, and assessment of their psychological health is discussed as integral to the transition program; however, TR data is not presented. Readiness was determined by the pediatric hepatologist, transition nurse, and the patient based on cognitive development, rather than chronological age, although the median age at transfer (18.6 years) was prior to more mature EF. The role of the transition specialist nurse that spans both pediatric and adult care and the effect of that continuity of care that extends into the adult setting for several years would be of interest as a factor that supports engagement in adult care [75].

**TABLE 4:** Outcomes after transfer to adult care in liver transplant.

Reference	Population	Outcome
Annunziato et al. 2007	Retrospective review of LT recipients (*n* = 14) to examine changes in adherence post‐transfer	Medication adherence significantly decreased after transfer compared to a cohort of YA within pediatrics; four patient deaths. Transfer occurred within the same institution with some continuity between adult and pediatric clinic; no specific TR interventions or a TOC program were utilized.
Sagar et al. 2015	Retrospective review of LT recipients transferred to adult care from 1994 to 2012 (*n* = 137)	Post‐transfer 10‐year patient/graft survival was 89.9% and 86.2%, respectively. Good long‐term outcomes reported in regard to clinical data; transition interventions inconsistent as to provider and strategies. TR data not presented. Endorses a transition specialist RN to span pediatric and adult care.
Rajchert et al. 2016	Retrospective review of LT recipients transferred to adult care from 2000 to 2015 (*n* = 20; median follow‐up 12.2 years)	Patient survival was 90%. At least 1 episode of ACR was reported in 75% of patients after transfer; usually due to nonadherence Patients transferred following a final “careful” checkup and given a referral letter, medication list, and adherence evaluation. No structured transition program was discussed. Concludes that a dedicated service for YAs may be needed to optimize outcomes.
Gabriel et al. 2017	Systematic review of transition studies in the chronic health literature (includes LT).	Positive outcomes reported in 65% of studies with a structure TOC program. Improvements reported in health maintenance, the transition experience, barriers to transition, resource utilization, and adherence.
Mitchell T et al., 2017	Clinical outcomes of LT recipients transferred to adult care (*n* = 18; mean age 18.2 years at transfer; mean follow‐up 4.7 years).	1‐ and 5‐ year survival of 100% and 92% respectively. Survival rates and adherence (66.7%) maintained after transfer; clinical data, adherence, and risk‐taking used as determinants of good outcomes. Transition interventions more administrative than educational.
Ferrarese et al. 2017	Outcomes report of LT recipients after TOC program and transfer (*n* = 32; median follow‐up 29 months)	Reported good long‐term clinical outcomes: no significant change in graft function; chronic rejection 18%, ACR 3%, nonadherence 25%, tobacco use 24.2%, ETOH use 15.6% and significantly associated with nonadherence. Education interventions resulted in improvements in mood (*p* < 0.001), emotional stability (*p* < 0.05), self‐awareness (*p* < 0.05), and a trend in improved adherence. Nonadherent patients with lower scores in self‐advocacy and self‐management.
Lawrence et al. 2020	Retrospective review of LT recipients who transferred to adult care 2002‐2016 (*n* = 32; median age at transfer 22.9 years).	Outcomes after transfer prior to a formalized transition program: mortality rate of 28%, retransplant required in 28%, significant decrease in office visit adherence (*p* = 0.02), and a trend toward worse health outcomes, episodes of ACR, and increased hospital admissions rate. Within the deceased group, there was a high rate of psychiatric co‐morbidity (67%) and substance abuse.

Culnane et al. 2021	Evaluation of a structured TOC program in LT from 2013 to 2015 (*n* = 28).	A structured transition program resulted in significant improvements in self‐advocacy skills in asking questions about their health, responsibility for medications, having insurance coverage, understanding risks of ETOH and drugs. All patients reported satisfaction with the program and 81% reported readiness for transfer.
Katz et al. 2021	Retrospective review of LT recipients who transferred to adult care from 1990 to 2015 (*n* = 64) without a structured TOC program.	Post‐transfer mortality rate of 28% with 55% mortality within 5 years after transfer; 8% lost to follow‐up. African Americans had a higher rate of mortality after transfer (44% vs 16%; *p* = 0.032).
Shapiro et al. 2021	Evaluation of a structured TOC program in LT using quality improvement methods and the Six Core Elements of Transition to measure processes (*n* = 24).	A structured TOC program resulted in significantly improved TR scores prior to transfer (*p* = 0.009) and improved adherence (45% vs 72%); 63% seen in adult care within 6 months of the last pediatric clinic.
Stevens et al. 2022	Retrospective study of LT recipients transferred to adult care from 2000 to 2015 (*n* = 101).	Prior to initiation of TOC program, a 24.7% mortality rate with 8% lost to follow‐up was reported. Associated factors included black race, psychiatric illness or substance use, failure to graduate prior to transfer, rejection, nonadherence, and having additional comorbidities (diabetes, CKD)

Another study of LT recipients transferred to adult care also reported that survival rates were maintained after transfer with 1‐ and 5‐year survival rates of 100% and 92% respectively [76]. Similar to Sagar et al. [75], the mean age at transfer was 18.2 years with a mean follow‐up time of 4.7 years after transfer. The clinical aspects of patient and graft survival were the determinants of good outcomes, and no late episodes of rejection were reported [76]. Readiness skill acquisition was not presented as a factor in successful transition; however, adherence and risk‐taking were assessed. Medication adherence (66.7%) was reported to be unaffected by transfer as evidenced by a MLVI of < 2 for those who had an adequate number of levels (*n* = 7) and when matched with other patients during the transition period [76]. The adherence rate for clinic attendance was 61%, and of the patients who did not attend clinic as requested, 71.4% had documented psychological issues. Risk‐taking behaviors and incidence in this cohort included alcohol, tobacco and illicit drug use at 26.6%, 15.4%, and 15.4%, respectively. Prior to transfer, 26.7% of youth had a psychiatric diagnosis, and an additional patient was diagnosed with an anxiety disorder post‐transfer [76]. Interventions of this TOC program appeared to be more administrative rather than educational, and included a verbal and written summary between the pediatric and adult LT provider, appointment scheduling by a clinical nurse consultant to provide overlap between the two setting during the transition year, and attendance by a pediatric nurse at the first adult clinic visit.

A similar outcomes report of young adult LT cites good long‐term outcomes after transition to adult care (*n* = 32) [71]. Although transition interventions were not detailed, transition begins with the pediatric transplant hepatologist and an educator assessing the YA for “self‐awareness” of their medical condition and medications, and having a “good” relationship with providers in clinic during the follow‐up period. At a median follow‐up of 29 months post‐transfer to adult care, no significant changes in liver function tests were observed, although the incidence of chronic rejection was 18%. There was one episode of acute rejection due to nonadherence that was successfully treated. Nonadherence to immunosuppressive therapy was 25%, and patients who reported to be nonadherent had a significantly higher MLVI than those who self‐describe as adherent (1.84 vs. 1.1; *p* = 0.04). Alcohol use was reported at 15.6% and was significantly associated with nonadherence (*p* = 0.03); 24.2% of patients reported using tobacco. Education was an important factor in this transition program with the transplant educator facilitating educational interventions that pertained to life and transition skills, and quality of life. A subsequent pilot study pre‐test that included 43% of the original study group identified emotional stability, mood, couple relationship, and resistance to stress as the most impaired domains of quality of life [71]. Lower scores were seen in nonadherent patients in regard self‐advocacy and self‐management. Although interventions were not detailed, educational strategies resulted in improvements in mood (*p* < 0.001), emotional stability (*p* < 0.05) and self‐awareness (p < 0.05) and a trend in improved adherence was appreciated [71].

In contrast to the findings of Ferrarese et al. [71] who reported lower self‐advocacy scores in nonadherent patients, significant improvement in self‐advocacy skills were demonstrated following interventions through a structured transition program for LT recipients [46]. Skills were characterized by the YA's ability to ask questions about their health, being responsible for medical equipment and supplies (prescriptions and ordering), having insurance coverage, and understanding the effects of drugs and alcohol [46].

Although positive outcomes are reported after transfer, the risks and incidence of nonadherent behaviors, graft dysfunction, and death highlight the need for comprehensive HCT services that engage the youth and providers. Decreased medication adherence, an increased incidence of rejection due to nonadherence, and graft loss and death have been demonstrated in young adult LT recipients after transfer to adult care [2, 4, 20, 77]. Outcomes prior to the initiation of a structured TOC program in LT were concerning as described in a retrospective study of young adult LT recipients who were transferred to adult care in 2000–2015 [5]. A mortality rate of nearly 25% with an additional 8% of patients as lost to follow‐up was reported in the absence of transition interventions. Several factors present in the pediatric care setting that were significantly associated with death after transfer to adult care including black race, psychiatric illness or substance use, failure to graduate from high school prior to transfer, nonadherence, incidence of rejection, and having additional comorbidities of diabetes and chronic kidney disease [5]. Similar outcomes were described in a retrospective review of LT recipients who had transferred care to adult providers from 2002 to 2016, prior to having a formalized transition program, comparing data from 2 years prior to 2 years post‐transfer [3]. A mortality rate of 28% following transfer to adult providers was reported and 28% required retransplantation. Although not reaching significance, the study reported a trend toward worse health outcomes, decreased adherence, and increased health care utilization following transfer of care. Additionally, there was a high rate of psychiatric co‐morbidity (67%) and substance abuse within the group of patients who died post‐transfer. The study endorsed the need to identify factors and interventions that support transfer of care and an improved process [3].

## 9 Transfer to Adult Care: Ongoing Transition in the Adult Setting

It is important to remember that transition is a process and that the timing of transfer of care should not be based on chronological age. Instead, the timing of transfer should be individualized and determined by TR, disease stability, availability of adult providers to engage in care, and other life circumstances. Additionally, TOC does not end at transfer but extends into adult care until the young adult has achieved optimal EF to the best of their ability. The Six Core Elements of Health Care Transition have been adapted for use in the adult setting after transfer to provide guidance for continuing HCT after transfer by the adult transplant hepatology team [38, 78]. The young adult should have more frequent contact with adult providers immediately after transfer to monitor for potential gaps in care, nonadherence to medications and lab requests, and to engage them through clear, consistent communication. The YA may need additional education that reinforces previous learning in regard to reproductive health, risk‐taking behaviors, and long‐term transplant related co‐morbidities. The partnership between the adult and pediatric providers should continue should questions arise from providers or the YA. Both short‐ and long‐term outcome measures should be assessed and root cause analyses performed for poor outcomes [33, 38, 78].

## 10 Summary and Future Implications

Recent position statements in LT, as well as other SOT, impart a greater awareness of the importance of transition of care within the transplant journey of the patient and family, and the impact of effective transition for long‐term graft survival and psychosocial outcomes. As discussed in this review, there is broad agreement among center‐specific studies and organizations that reflect a similarity in approach, but standardization with consistent outcomes measurement is lacking. The literature supports transition as an essential component of care in transplantation that should be initiated in early adolescence. An assessment of transition readiness, rather than chronological age, should be the primary determinant of transfer. TOC programs should address LT knowledge, skill development, and self‐advocacy, and provide an efficient process that helps youth engage in their care through a supportive environment and in collaboration with the pediatric and adult center. The engagement of all stakeholders in LT is essential to develop and sustain a structured transition program that will assist youth in attaining self‐management skills. There is greater awareness of the adult provider role in TOC, and that transitional care must extend into the adult setting as EF skills mature. Relationship barriers between the youth, parent, and providers may present challenges throughout the process, but can also provide opportunities to strengthen new relationships built on the foundation of previous relationships and support stakeholders in their new roles. There is a critical need for adult providers to care for the increasing population of YAs with childhood‐onset chronic liver disease and LT who are surviving into adulthood and transitioning to adult. Limits in willingness to accept these patients are often related to the adult providers' lack of training in pediatric congenital diseases, the patient's inadequate readiness for transition, and the relationship between the patient and the pediatric provider.

A standardized approach to HCT in liver transplant is needed and should be based on intervention studies to determine best practice and the effectiveness of transition tools. A framework for program development as detailed through the Six Core Elements of Transition provides tools and program guidance that are readily adaptable to LT. Education and training in transition services is needed for all providers within the multidisciplinary team not only to understand their role in the process but to promote their engagement in transition. Incorporating a transition curriculum within medical, nursing, and advanced practice training has been recommended [79]. Additional educational support is available through multidisciplinary learning health systems, such as the Starzl Network, which is committed to developing and disseminating best practices in LT and has identified TOC as a priority area of study [27].

Future efforts and progress in health care transition will require changes in health care policy supported through institutional and organizational outcomes research that demonstrates the effectiveness of transition interventions and the implications on long‐term outcomes. Outcome measures in LT need to be clearly defined and standardized and should reflect not only patient outcomes but the effect of inadequate transition on health care utilization and costs. Regulatory agency involvement to validate and support the need for TOC programs is crucial and should promote outcomes research for best practice program standardization.

## Conflicts of Interest

Nitika Gupta advises Albireo and Alexion. The remaining authors declare no conflicts of interest.

## Data Availability

Data sharing not applicable to this article as no datasets were generated or analyzed during the current study.
